# Single-cell sequencing reveals transcriptional dynamics regulated by ERα in mouse ovaries

**DOI:** 10.1371/journal.pone.0313867

**Published:** 2024-11-21

**Authors:** Qicai Hu, Yiqian Gui, Congcong Cao, Jun Xie, Huiru Tang

**Affiliations:** 1 Center of Obstetrics and Gynecology, Peking University Shenzhen Hospital, Shenzhen, China; 2 Institute of Obstetrics and Gynecology, Shenzhen PKU-HKUST Medical Center, Shenzhen, P. R. China; 3 Shenzhen Key Laboratory on Technology for Early Diagnosis of Major Gynecologic Diseases, Shenzhen, P. R. China; 4 Institute Reproductive Health, Tongji Medical College, Huazhong University of Science and Technology, Wuhan, China; 5 Guangdong and Shenzhen Key Laboratory of Reproductive Medicine and Genetics, Institute of Urology, Peking University Shenzhen Hospital, Shenzhen PKU-HKUST Medical Center, Shenzhen, China; 6 Cheerland Watson Precision Medicine Co. LTD, Shenzhen, China; University of Wisconsin-Madison, UNITED STATES OF AMERICA

## Abstract

Context: Estrogen receptor α (ERα) is a key regulator of reproductive function, particularly in ovarian development and function, yet the specifics of its role at the molecular level remain unclear. Aims: The study aims to elucidate the molecular mechanisms of ERα-regulated transcriptional dynamics in ovarian cells using ERα knockout (αERKO) mice created via CRISPR/Cas9. Methods: Single-cell RNA sequencing (scRNA-seq) was used to compare transcriptomes from individual ovarian cells in both wild type and αERKO mice. Bioinformatics analyses identified distinct cell populations and their transcriptional profiles post ERα deletion. Key Results: Distinct oocyte and granulosa cell populations were identified, with ERα deletion disrupting the regulation of genes linked to ovarian infertility, the ovulation cycle, and steroidogenesis. Greb1 expression in granulosa cells was found to be ERα-dependent. Conclusions: ERα deletion significantly alters the transcriptional landscape of ovarian cells, affecting genes and pathways central to ovarian function and the ovulation process. Implications: The findings provide an in-depth, single-cell view of ERα’s role in the reproductive system, offering insights that may lead to novel treatments for ovarian disorders.

## Introduction

Estrogen is a steroid hormone that is well-known for its role in female reproduction. It is synthesized in the ovaries and is essential for controlling the estrous or menstrual cycle in females [1]. The primary mechanism of estrogen action is through the expression of nuclear estrogen receptors (ER) in estrogen target organs. The biological effects of estrogen are mediated through two distinct ER proteins, ERα (ESR1) and ERβ (ESR2), which are encoded by separate genes on different chromosomes and have different expression profiles in tissues [2]. ERα is present in all three components of the hypothalamic-pituitary-ovarian axis of the mouse. In contrast, ERβ is easily detectable in ovarian granulosa cells but is low to absent in the pituitary of the adult mouse. This distinct expression pattern for the two ERs suggests the presence of separate roles for each in the regulation of ovarian function. In mammals, the ERs are members of the nuclear receptor superfamily of hormone-inducible transcription factors [3, 4]. Like other nuclear receptors, they work in the nucleus of cells and are receptors for specific hormones. All members of the nuclear receptor family share a multidomain structure, with each domain directing the mechanistic interactions and functions necessary for hormone response. The ERs have six domains, A through F [5]. The ligand-binding domain (LBD) and DNA-binding domain (DBD) are critical domains for estrogen receptor activity. In addition, the LBD is responsible for high-affinity and high-specificity binding to its hormonal activator estrogen and the DBD is responsible for binding to the estrogen-responsive element (ERE), a target gene DNA motif. Each domain, including the LBD and DBD, encodes structural features that are essential for the receptor’s activity [6, 7].

Gene targeting technology was used to generate mice strains with disrupted ERα (αERKO), ERβ (βERKO), and a compound αβERKO throughout their entire body. Male and female αERKO mice are sterile. Despite treatment with estrogen, EGF, and IGF-1, αERKO uteri fail to undergo DNA synthesis and uterine growth. Female αERKO mice are infertile due to hypoplastic uteri and hyperemic ovaries that lack corpora lutea, a consequence of persistent LH stimulation from the loss of negative feedback [8]. Male αERKO mice are also sterile, with testicular atrophy and seminiferous tubule dysmorphogenesis that result in reduced spermatogenesis and inactive sperm. Similar pathological disorders were observed in the aromatase knockout mice, demonstrating a critical role of ERα-mediated estrogen action in regulating ovarian function [9]. In contrast, βERKO females exhibit arrested folliculogenesis and subfertility, with ovarian analysis revealing differential gene expression patterns associated with ovulatory stimulation deficits, including the absence of LH, PR, Cyp19, and Cox2 expression [10, 11]. In addition, a unique ovarian phenotype is observed only in αβERKO females, characterized by the transdifferentiation of granulosa cells into Sertoli cells [12]. Finally, another study indicates that ERα, but not ERβ, is indispensable to the negative-feedback effects of estradiol that maintain proper LH secretion from the pituitary. Therefore, the ERα knockout mouse provides a unique tool to clarify the role of estrogen receptor in ovarian function. And the αERKO ovarian phenotype may be the result of a lack of ERα -mediated action either within the ovary and/or at the level of the hypothalamic-pituitary axis.

Despite recent advances in understanding the biological responses mediated by ERα, no studies have investigated the impact of ERα on the development of oocytes and granulosa cells, and the molecular mechanisms involved in their interactions. The ovary contains different cell types with distinct states and interactions, and gene expression patterns can vary greatly across these cell types under different conditions. Most ovarian studies have relied on whole ovaries or follicles, making it difficult to detect changes in gene expression of specific cell types within the ovary. This is because bulk RNA-seq compares the average gene expression levels of all cells, making it challenging to explore differences at the cellular level. However, single-cell RNA sequencing (scRNA-seq) has emerged as a promising tool for studying gene expression and cellular diversity in various tissues, including the ovary [13–15]. For example, Fan et al. conducted a transcriptome analysis to document the changes that take place during the process of follicular development and regression using human ovary surgical specimens [16]. In order to examine the alterations in cell types and states that are associated with aging, a primate model was employed [15]. In a separate study, Zhao et al. investigated the early embryonic ovarian development, specifically focusing on the relationship between oocytes and their supporting cells in the formation of follicles [13]. This demonstrates that we can use scRNA-seq to investigate different physiological processes and provide insights into the molecular mechanisms by which ERα impacts ovarian cells.

In the present study, to understand more fully the effects of ERα on ovarian cells, we performed high-throughput scRNA-seq analysis of ovaries from adult αERKO and control mice. The results and bioinformatics information reported here will certainly be useful for elucidating the molecular mechanisms by which ERα affects female germ cells and granulosa cells during follicular development and ovulation, and the transcriptional programs associated with ovarian suppression by ERα.

## Methods

### Animals

Wild-type (WT) mice and ERαKO mice in C57/B6J background were established through CRISPR-Cas9 strategy from Cyagen company. Genotyping was performed using mouse tail genomic DNA, and PCR with primers as indicated. All animals used in this study were maintained in SPF laboratory animal room and treated according to the Guide for the Care and Use of Laboratory Animals prepared by the Institute of Laboratory Animal Resources for the National Research Council. This study was approved by the ethics committee of Peking University Shenzhen Hospital and the animal center of Shenzhen PKU-HKUST Medical Center.

### Histology of ovary

The ovaries of adult female mice were extracted from both control and ERKO mice, and then fixed in 4% paraformaldehyde (PFA) overnight. The tissues were subsequently embedded in paraffin, sectioned into slides, and stained with Hematoxylin and Eosin (HE) using previously established methods. After sealing with neutral gum, microscope images were captured using an Olympus BX53 microscope located in Tokyo, Japan.

#### Methods of sacrifice

At the end of the experiment, all mice were humanely euthanized by CO₂ asphyxiation followed by cervical dislocation to ensure death. This method was chosen as it is considered a rapid and humane method of euthanasia, compliant with the AVMA Guidelines for the Euthanasia of Animals.

#### Methods of anesthesia and/or analgesia

For procedures that required the handling and manipulation of animals, including surgeries, the mice were anesthetized with isoflurane (1–2% in oxygen) delivered through a precision vaporizer. Isoflurane was chosen due to its rapid onset, controllability, and fast recovery profile, minimizing stress to the animals.

#### Efforts to alleviate suffering

To minimize potential pain and distress, mice were monitored closely during and after the procedures. Analgesics, such as meloxicam (5 mg/kg), were administered preemptively and postoperatively as required to control pain. Additional care was taken to ensure that the animals were provided with a comfortable environment, with appropriate bedding, hydration, and nutrition, to reduce stress and promote recovery.

### IHC

Ovarian tissues were obtained from 12-week-old female mice, both WT and ERα KO, and subsequently embedded in paraffin. Paraffin-embedded sections (5 μm) were prepared on glass slides and underwent deparaffinization and hydration. Antigen retrieval was performed by heating slides in 0.01 M sodium citrate buffer (pH 6.0) for 15 min, followed by cooling to room temperature and washing thrice with PBS for 5 min each. Endogenous peroxidase activity was quenched using 3% hydrogen peroxide in PBS for 10 min. Non-specific binding was blocked by incubating with 10% lamb serum in PBS for 1 h at room temperature. Subsequently, sections were exposed to the primary antibody at 4°C overnight. Antibodies against RHOX8 (Abcam, ab237009), GREB1 (Abcam, ab72999), CYP19A1 (Abcam, ab106168) and IGF1 (Abcam, ab263903) were used to detect each protein at a dilution factor of 1:200 each. After PBS washes, sections were incubated with biotinylated goat anti-rabbit IgG secondary antibody (1:200 dilution, Invitrogen, USA) for 1 h at room temperature, using 10% lamb serum in PBS as the dilution buffer. Immunoreactive signals were visualized using streptavidin-HRP and VECTOR Nova RED Peroxidase (HRP) Substrate Kit (Vectorlabs, Burlingame, USA) at room temperature. Hematoxylin counterstaining was performed, and negative controls were processed without primary antibody incubation. Immunostaining was examined using a Nikon Eclipse 50i microscope (Nikon, Tokyo, Japan) and documented with NISElement F Software. Each antibody experiment was repeated using slides from three distinct ovaries of the same genotype.

### Isolation of ovarian cells

A two-step enzymatic digestion protocol, which had been previously described [17], was utilized to isolate single cells of ovary. Ovaries from adult control or ERKO female mice were first dissected and decapsulated in PBS. Collagenase Type IV (Sigma-Aldrich, V900893) was added to the tissues and incubated at 37°C for 5 min with gentle agitation. The tissues were then separated and washed twice with PBS before being subjected to digestion with trypsin (Gibco, 15090046) and DNase I (Sigma-Aldrich, DN25) at 37°C for 20 min with periodic vigorous agitation. The cells were filtered through strainer and centrifuged at 600 g for 5 min at 4°C. The dissociated cells were subsequently washed twice with PBS and used for scRNA-seq.

### 10x genomics library preparation

The procedure involves creating a single-cell suspension sample, counting and measuring the viability of cells using the CountessII Automated Cell Counter. The Cell activity needs to be over 80% and the cell concentration should be adjusted to 1000/μL. The single-cell suspension is then mixed with gel beads containing barcode information and enzymes and encapsulated in microfluid droplets to form gel bead-in-Emulsions (GEMs). Effective GEMs include gum beads, single cells, and Master Mix. Cell lysis and reverse transcription reaction take place in GEMs, followed by PCR amplification using the cDNA as a template. Quality inspection of amplified product is conducted before constructing the sequencing library. The cDNA is fragmented into 200-300bp fragments, followed by screening, and the P7 Adaptor connector is attached along with sample Index using PCR amplification. The cDNA library is obtained by fragment screening. After completion, the database is checked, and Illumina HiSeq sequencing platform is used to obtain the sequencing data, which is subsequently analyzed.

### scRNA-seq data processing

FastQC was utilized to conduct basic statistical analyses on the quality of the original read fragments. Next, the Trimmomatic software was employed to preprocess the FASTQ-formatted Illumina pipeline read fragment sequence, as outlined below: firstly, remove low-quality read fragments by scanning with a 4-base wide sliding window and trimming when the average base quality drops below 10. Secondly, remove trailing low-quality fragments or N bases (mean quality score <3). Thirdly, delete the adapter sequence using one of two methods: align and remove matches with a length >7 and mismatches equal to 2, or delete non-overlapping parts when the overlap base between read fragments 1 and 2 exceeds 30. Fourthly, fragments less than 26 bases in length were deleted. Finally, unpaired reads were discarded. The remaining reads that passed all filtering steps were considered as clean reads, and all subsequent analyses were conducted using these reads. Overall, basic statistics on the quality of the clean reads were successfully generated using FastQC.

### PCA and t-SNE analysis

To simplify the gene expression matrix by focusing on its most important features, we employed Cell Ranger’s PCA to change the data set dimension from (Cell x gene) to (Cell x M), where M refers to the principal component selected by the user. Our reanalysis pipeline enables us to further reduce the data by randomly resampling the cells and/or selecting genes across the data set in a dispersed manner. To visualize the two-dimensional spatial data, the author used Cell Ranger to transfer the data to the T-SNE nonlinear dimension reduction method after performing PCA dimension reduction. Additionally, we reduced the running time by fixing the number of output dimensions to 2 or 3 during compilation.

### Identification of specific genes in different cell clusters

In this study, we collected two ovaries each from two WT and two ERα KO mice. Ovaries from each group (WT and KO) were separately digested, and cells from all the tissues were processed together for single-cell RNA sequencing (scRNA-seq). It is important to note that the cell proportion statistics were calculated based on the pooled samples within each group (WT and KO) rather than separately digesting and analyzing the ovaries from individual mice. As a result, the cell proportions represent the overall group-level outcome, and no independent sample statistical tests (such as p-value calculations) were performed to account for biological variance.

To identify genes that have specific expression patterns in certain clusters, we tested each gene and cluster using Cell Ranger to compare the mean expression within the cluster to the mean expression outside of the cluster. To identify genes with differential expression between clusters, we used Cell Ranger’s sSeq method, which employs a negative binomial precision test. When the counts were high, we switched to the fast asymptotic beta test in edgeR, which was run on each cluster and compared to all other cells. This generated a list of genes that were differentially expressed in each cluster compared to the rest of the cells. We also used Cell Ranger to calculate the relative library size by dividing the total UMI count per cell by the median UMI count per cell. Normalization was implicit, as the parameter of each cell library size was combined as a factor in the precise test probability calculation, similar to sSeq.

### Enrichment analysis of KEGG pathway and GO function of differentially expressed genes

The differential expression of genes was subjected to enrichment analysis using KEGG and GO through the Profiler R package for cluster analysis, which was able to correct for gene length bias. Any KEGG pathways or GO terms showing a corrected P value of <0.05 (FDR<0.05) were deemed to be significantly enriched in the differentially expressed genes. Furthermore, the author employed gene MANIA in Cytoscape 3.6 to expose the interaction network between KEGG pathways and genes in GO functional annotation.

### Quantitative RT-PCR (RT-qPCR)

RNA was extracted from both WT and ERKO mice using Trizol reagent as per the manufacturer’s instructions. The extracted RNA was subsequently reverse transcribed into cDNA with the PrimeScript RT Master kit (Takara, RR037A). RT-qPCR was conducted using the SYBR® Premix EX TaqTM II PCR Kit (Takara, DRR041A) according to the manufacturer’s instructions on the Roche Lightcycler 480 Real-Time PCR System. The data obtained were analyzed using the comparative Ct method by Applied Biosystems [8, 18, 19]. The primers specified in S1 Table.

### Granulosa cells isolation, culture, and E2 treatments

Granulosa cells were obtained from wild-type (WT) and estrogen receptor alpha knockout (ERαKO) mouse ovaries, as previously described [20]. These cells were subsequently cultured in α-minimum essential medium (α-MEM; Invitrogen, USA) supplemented with 10% fetal bovine serum (FBS; Gibco, Thermo Fisher Scientific, USA). Prior to 17β-estradiol (E2) exposure, the cells were maintained for 24–48 hours in estrogen-deprived medium consisting of phenol-red-free Dulbecco’s Modified Eagle Medium/Nutrient Mixture F-12 (DMEM/F12; Invitrogen, USA), 1.2 g/L sodium bicarbonate, and 5–10% charcoal-stripped serum. E2 (Sigma, USA) was prepared in 100% ethanol and administered at a final concentration of 10 nM. For experiments investigating estrogen receptor 1 (ESR1) activation, the selective ERα agonist propyl pyrazole triol (PPT) was introduced to the culture medium.

### Luciferase reporter assays

In the present study, the firefly luciferase reporter vector containing the ERE sequence (3xERE-TATA-LUC; provided by Addgene LGC Standards, Teddington, UK) was utilized. The Renilla luciferase gene, regulated by the TK promoter (Promega), served as a control plasmid and was co-transfected to account for fluctuations in transfection efficiency. Granulosa cells were plated in 96-well plates at a density of 1.7 x 10^4^ cells per well using 10% CD-FBS PR-free DMEM/F12 media and allowed to adhere for 24 hours for transient transfection assays. Subsequently, both the reporter and control vectors were introduced into the cells for 6 hours employing Lipofectamine 2000 (Thermo Fisher Scientific) in Opti-MEM medium (Thermo Fisher Scientific), adhering to the supplier’s guidelines. The media was then replaced with fresh 10% CD-FBS PR-free DMEM/F12 medium. Following a 24-hour incubation period, cells were exposed to either vehicle or various pharmacological agents for an additional 24 hours. At the conclusion of the experiment, cell lysis was performed using Passive Lysis Buffer (Promega). The luciferase activities were quantified with the Dual Luciferase Reporter Assays System (Promega) and a Berthold Lumat LB9507 luminometer (Berthold France, Thoiry, France). The firefly luciferase-derived transactivation activity was normalized to that of Renilla luciferase.

### Chromatin immunoprecipitation

Granulosa cells underwent treatment with 100 nM 17β-estradiol (E2) for a duration of 45 minutes, followed by crosslinking using 1% formaldehyde, subsequent quenching with 125 mM glycine, and sonication to generate DNA fragments ranging from 150 to 200 base pairs. An ESR1-specific antibody (Santa Cruz Biotechnology) and a normal mouse IgG control (Millipore, USA) were coupled to magnetic beads (Dynabeads; Thermo Fisher Scientific, USA) through an overnight incubation at 4°C. Subsequently, 250 μg of DNA underwent preclearing with the addition of 1 μl/ml salmon sperm DNA (Sigma, USA), 10 μl/ml ovalbumin (Sigma, USA), and 10 μl/ml magnetic beads, with incubation for 1 hour at 4°C. A 10% aliquot of the precleared chromatin was reserved as the "input" fraction, and the DNA was immunoprecipitated with the antibody-coated beads overnight at 4°C. The immune-complex-bound beads were collected using a Magna GrIP Rack (Millipore, USA) and eluted at 65°C for 10 minutes using an Eppendorf Thermomixer. Each sample underwent reverse crosslinking and protease treatment. Genomic DNA fragments obtained through immunoprecipitation were purified using a phenol:chloroform:isoamyl alcohol extraction method. The mouse *Greb1* ERE promoter regions, encompassing potential ERE, were amplified utilizing the HotStarTaq DNA Polymerase Kit (Qiagen, USA) and the primers specified in S1 Table.

### Statistical analysis

The results were presented as the mean ± standard deviation (SD). Statistical analyses were performed using GraphPad Prism 8.0 software (GraphPad, San Diego, CA, USA) and SPSS 20.0 (IBM, SPSS, Chicago, IL, USA). For the comparison between two groups, the two-sided Student’s t-test or the two-tailed Mann-Whitney U-test was employed, depending on the data distribution. When comparing multiple groups, one-way analysis of variance (ANOVA) followed by Bonferroni post hoc tests was utilized. Statistical significance was considered at a P-value of less than 0.05. And each of the experiments mentioned were conducted with a minimum of three technical replicates.

## Results

### scRNA-seq identified cell population types within ovaries of control and αERKO mice

**Fig 1:**
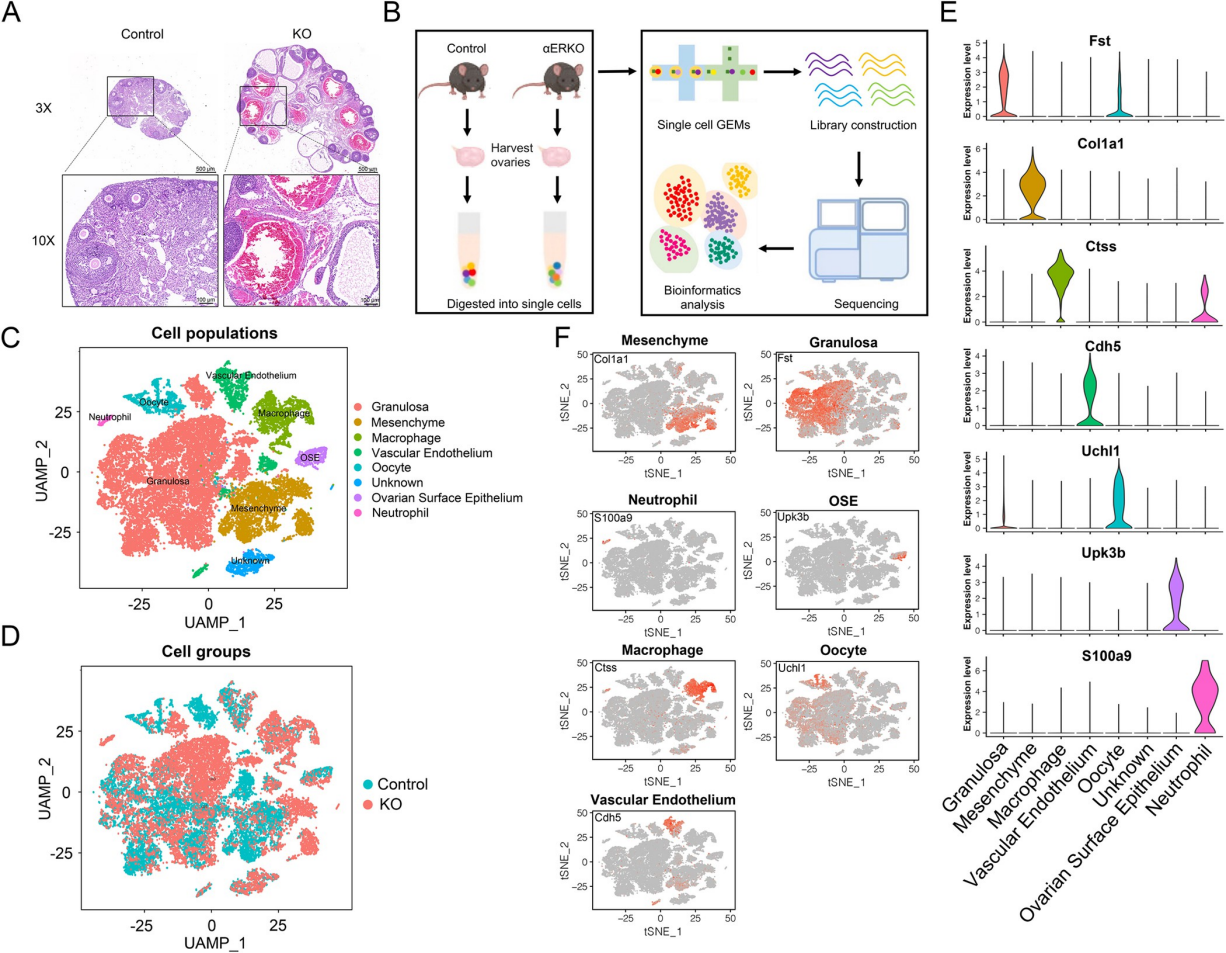
Single-cell RNA sequencing of control and ERα KO mouse ovaries. (A) Hematoxylin and eosin (H&E)-stained sections of control and ERα KO mice ovaries. (B) Schematic diagram illustrating the experimental pipeline for scRNA-seq analysis of control and ERα KO mice ovaries. Single-cell transcriptomes were obtained based on the 10 × Chromium platform. (C-D) Uniform manifold approximation and projection (UMAP) plot featuring the different clusters of the ovary (C) and their composition by control and KO mice (D). (E) Violin chart showing the expression of specific marker genes in different cell types. (F) Expression of marker genes on UMAP map in different cell types.

To determine the function of ERα in mouse ovary, we firstly generated ERα knockout mice using CRISPR/Cas9 strategy. The results of hematoxylin-eosin (HE) staining revealed that αERKO mice exhibited enlarged, hemorrhagic, and cystic follicles in their ovaries. In addition, adult KO females were also anovulatory, possessing pre and small antral follicles, but lacking corpora lutea, which led to infertility (Fig 1A and S1E Fig). In order to investigate the transcriptional dynamics of ovaries at a single-cell resolution, we then conducted the scRNA-seq involving adult control and αERKO (KO) mice. After isolating the ovaries and performing enzymatic digestion, we obtained single-cell suspensions which were sorted using microfluidics. Subsequently, we prepared indexed libraries for sequencing (Fig 1B).

To investigate the heterogeneity of ovarian cells, we conducted t-distributed stochastic neighbor embedding (tSNE) analysis on high-quality cell populations, followed by Uniform Manifold Approximation and Projection for Dimension Reduction (UMAP) and Principal Component Analysis. Subsequently, we integrated control and KO samples, resulting in the identification of 14 cell clusters (S1A Fig). Based on the heatmap results of these cell clusters, we classified the entire cell population into eight cell types, which included granulosa cells, mesenchyme cells, macrophage cells, vascular endothelium cells, oocyte, an unknown cell type, ovarian surface epithelium cells (OSE), and neutrophils (S1B Fig and Fig 1C). Further clustering of the cell populations at control and KO samples was performed using UMAP analysis (Fig 1D). The expression of specific genes known to be associated with each cell type was examined, and representative genes such as *Col1a1*, *Fst*, *S100a9*, *Upk3b*, *Ctss*, *Uchl1*, and *Cdh5* were used to label mesenchyme cells, granulosa cells, neutrophils, OSE cells, macrophage cells, oocyte, and vascular endothelium cells, respectively (Fig 1E and 1F). The percentage of cell counts for each cell type in the control and KO samples was presented in S1C Fig. In order to further characterize the transcriptional signatures of the identified cell types, a heatmap of top gene expression across major categories of cell types was generated (S1D Fig). These analyses provided a preliminary delineation of the effects of ERα inactivation on the progression and dynamics of the ovarian cell lineage. Following, we focused on the differentiation fate of the major participants involved in follicular development and ovulation, specifically the oocyte and granulosa cells.

### High-resolution analysis of the effect of ERα disruption on oocyte

**Fig 2:**
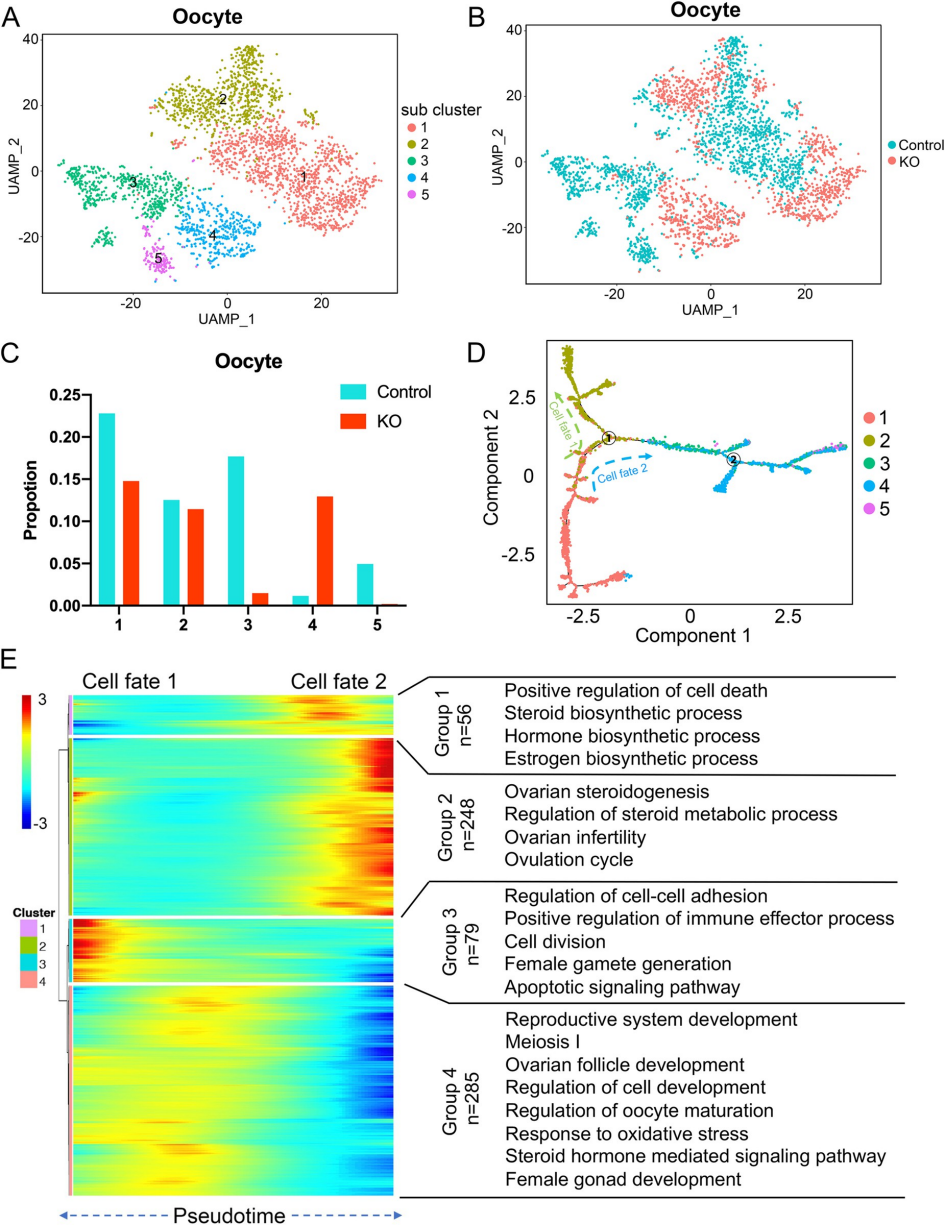
Analysis of the heterogeneity of oocyte subsets. (A) UMAP plot featuring the different cell subclusters belonging to the oocyte cluster. (B) UMAP diagram of the oocyte subpopulations of control and KO groups. (C) Proportion of the five cell subclusters of the oocytes in control and KO groups. (D) Single-cell pseudotime developmental trajectory of oocytes, which are colored according to cell development state. (E) The heatmap shows the gene expression changes of oocytes of four genomes in the two cell fate branches at point 1 (left). GO term enrichment results of the four gene sets (right).

To investigate the heterogeneity of oocyte populations following ERα inactivation, we performed UMAP analysis to subcluster oocyte populations from control and KO samples. We identified five distinct oocyte subclusters, as displayed in tSNE plots (Fig 2A and 2B). The top five expressed genes of each subcluster were examined, and a heatmap was generated (S2A Fig). We found that most oocytes in subclusters 3 and 5 were from control samples, while most oocytes in subcluster 4 were from KO samples. In subclusters 1 and 2, the numbers of control and KO oocytes were roughly equal (Fig 2C). To investigate the origin and maturation process of oocytes, we used Monocle to construct a pseudotime developmental trajectory, which revealed two branches in the oocyte lineage trajectory. Branch 1 was found to play a critical role in determining two different cell fates (Fig 2D). Notably, both the pseudotime development trajectory and the stacking diagram demonstrated that ERα deletion increased the number of oocytes that developed toward state 2 (subcluster 4) (refer to S2B Fig). We analyzed the time series of oocyte development while eliminating cell cycle effects from the oocyte clusters (S2D and S2E Fig). Using the pseudotime development trajectory, we compared changes in gene expression at the two branches and identified four different gene sets (Cluster1: 56; Cluster2: 248; Cluster3: 79; Cluster4: 285) whose expression changed significantly along with the cell trajectory (Fig 2E). We employed GO enrichment analysis to study the gene function of these differentially expressed genes (DEGs). The genes that were highly expressed at the starting and intermediate positions of oocyte developmental trajectory (group 4) were found to be involved in biological functions related to meiosis and ovarian follicle development. The genes highly expressed in oocytes at the end of cell fate 1 (group 3) were mainly associated with cell division, female gamete generation, and apoptotic signaling pathway. The genes that were highly expressed in oocytes at the intermediate positions of cell fate 2 (group 1) were more involved in biological functions such as steroid biosynthetic process. The genes highly expressed in oocytes at the end of cell fate 2 (group 2) were mainly associated with ovarian steroidogenesis, ovulation cycle, and ovarian infertility (Fig 2E). Our findings suggest that ERα disruption in the ovary could dysregulate steroid metabolic processes and prevent oocytes from completing the ovulation progression.

### Loss of functional ERα in the ovary alters the transcriptome of oocyte

**Fig 3:**
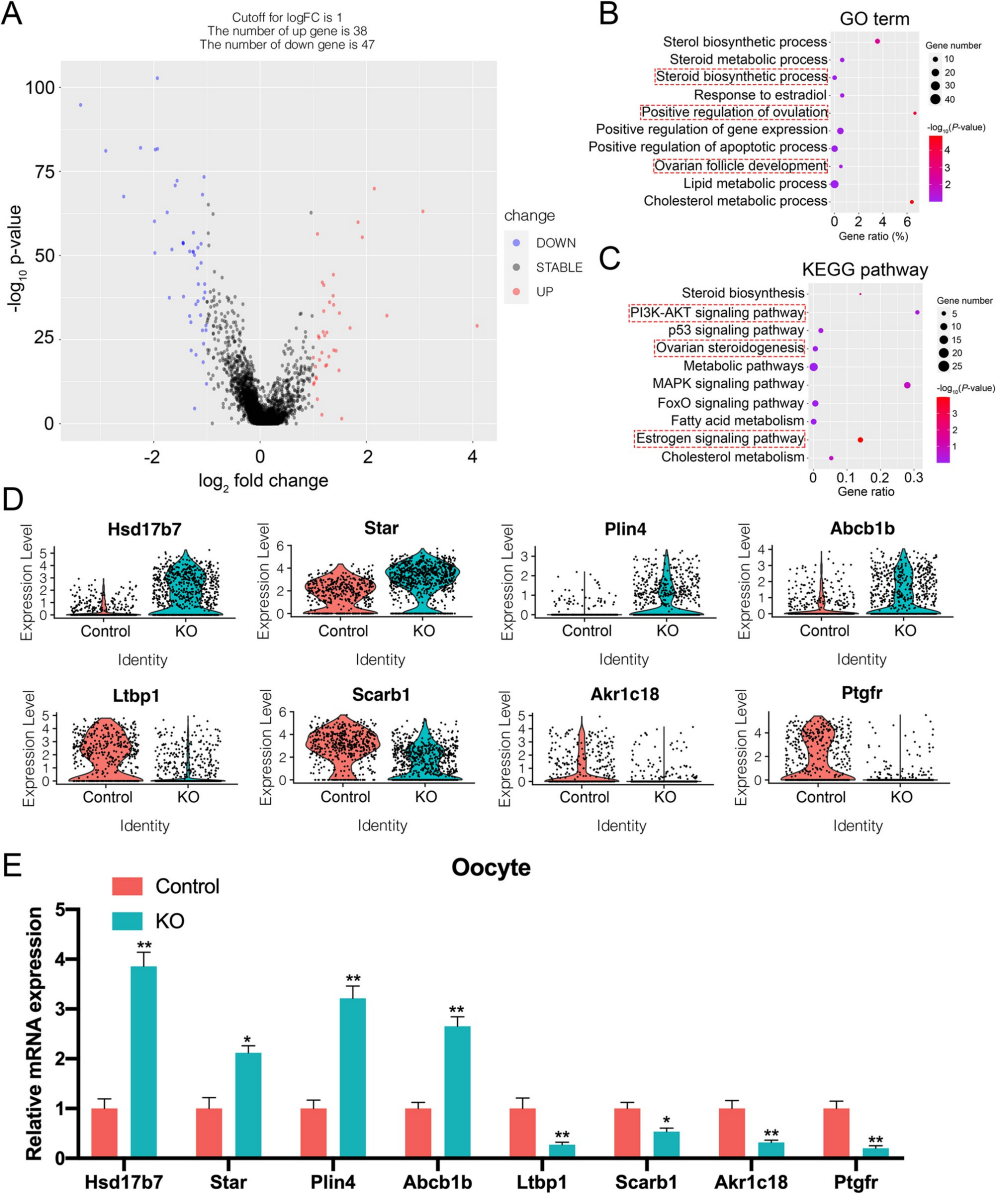
Analysis of DEGs of oocytes between control and KO groups. (A) Volcano plot of genes differentially expressed in oocytes between control and KO groups. (B) Bubble chart shows the GO enrichment results of differentially expressed genes in oocytes between control and KO groups. (C) KEGG enrichment results of DEGs in oocytes. Bubble chart shows the KEGG enrichment results of differentially expressed genes in oocytes between control and KO groups. (D) Vlnplots of the expression level of representative DEGs in oocytes between control and KO groups. (E) RT-qPCR validation of representative DEGs in oocytes between control and KO groups. *p < 0.05, **p < 0.01.

To elucidate the underlying mechanism of ERα disruption-induced female infertility in oocyte, we conducted a differential gene expression analysis between control and KO oocyte groups. We identified 38 upregulated and 47 downregulated genes with a |log2(FC)| > 1 in the KO group compared to the control group (Fig 3A and S2 Table). We performed GO enrichment and KEGG pathway analysis of the differentially expressed genes in oocyte. The GO terms related to "steroid biosynthetic process", "positive regulation of ovulation", and "ovarian follicle development" were significantly enriched in oocyte (Fig 3B). Moreover, the KEGG molecular pathways of oocytes were primarily enriched in "PI3K-AKT signaling pathway", "ovarian steroidogenesis", and "estrogen signaling pathway" (Fig 3C).The identification and analysis of DEGs in oocyte revealed that several upregulated genes involved in estradiol synthesis and steroidogenesis, including *Hsd17b7* (which plays a key role in estrogen biosynthesis), *Star* (which is essential for cholesterol transport and steroid hormone production), and *Plin4* (which is involved in lipid droplet metabolism crucial for steroidogenesis (Fig 3D and S1F Fig). *Abcb1b*, which has previously been reported to be LH responsive in the ovary, was also increased in KO oocyte (Fig 3D) [8, 18, 21, 22]. In contrast, the downregulated genes *Ltbp1* and *Akr1c18* have been shown to decrease female mouse fertility (Fig 3D) [23, 24]. The expression levels of *Scarb1* and *Ptgfr*, which play an important role in luteinization, were also significantly decreased in the KO group. Finally, we confirmed that the transcriptional gene expression changes were consistent between the scRNA-seq data and oocyte transcripts quantified by RT-qPCR (Fig 3E). Overall, our findings suggest that the evaluation of ovarian steroidogenic capacity and morphological aberrations of ovarian structures in αERKO female mice may be attributed to the dysregulation of these genes in the ovary.

### Dissecting the change in cell fates of granulosa cells affected by ERα disruption

**Fig 4:**
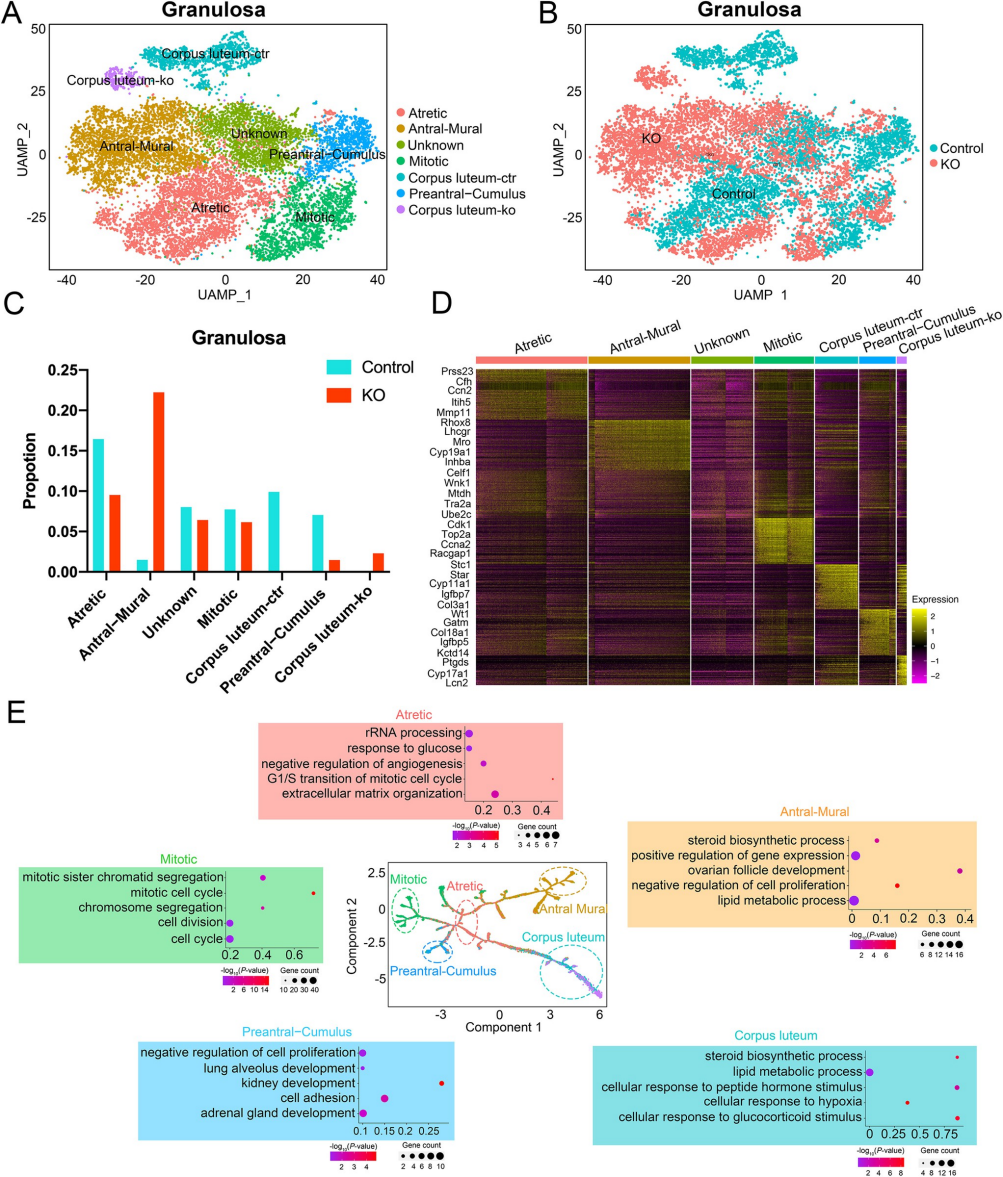
Identification of the different cell types in the granulosa cluster. (A) UMAP plot featuring the different cell subclusters belonging to the granulosa cluster. (B) UMAP diagram of the granulosa cell subpopulations of control and KO groups. (C) Proportion of the seven cell subclusters of the granulosa cells in control and KO groups. (D) Heatmap of the top five markers of each subcluster in granulosa cells by fold change. (E) Developmental trajectories of different granulosa cell subclusters in pseudotime. Representative GO terms for stage-specific genes are shown.

To investigate the cellular heterogeneity within developing follicles, we analyzed the subclustering of granulosa cells based on their transcriptional profile. We were able to distinguish discrete granulosa cell states in follicles based on their stage of development, consistent with previous reports [13, 16, 25]. We subdivided granulosa cells into seven main categories: atretic, antral-mural, unknown, mitotic, corpus luteum-control, preantral-cumulus, and corpus luteum-ko (Fig 4A). The control and KO groups were evenly distributed in the UMAP chart (Fig 4B). The percentages of cell count from different samples in each cluster were shown in Fig 4C. Our findings revealed that most mural granulosa cells of antral follicles were identified as KO cells (22.2%), while most preantral granulosa cells constituting the cumulus oophorus of antral follicles were identified as control cells (7%). In addition, the corpus luteum cluster could be found in both control (9.9%) and KO (2.3%) samples, but most of them were identified from control granulosa cells. We identified distinctive gene expression programs in the granulosa cell subclusters, as visualized in the heatmap, from which we selected potential markers for validation (refer to Fig 4D) [19]. To reconstruct the pseudotime trajectory of the granulosa cells during developing follicles, we performed Monocle analysis on subclustering of granulosa cells (S3A–S3C Fig). We observed that the granulosa cells from control and KO groups were distributed in different branches. Then we performed GO term enrichment analysis on the DEGs of the cells on the five subclusters and found that GO terms related to "steroid biosynthetic process", "positive regulation of gene expression", and "ovarian follicle development" were enriched in the antral-mural cluster. GO terms related to "rRNA processing" and "response to glucose" were enriched in the atretic cluster. GO terms related to "mitotic cell cycle" and "cell division" were enriched in the mitotic cluster. GO terms related to "cell adhesion" and "multiple tissues development" were enriched in the preantral-cumulus cluster. Finally, GO terms related to "steroid biosynthetic process" and "lipid metabolic process" were enriched in the corpus luteum cluster (Fig 4E). Our results suggest that the lack of ERα in the ovary influenced the development and function of granulosa cells, which play an important regulatory role in ovulation induction.

### Gene expression signatures of the granulosa cell lineage

**Fig 5:**
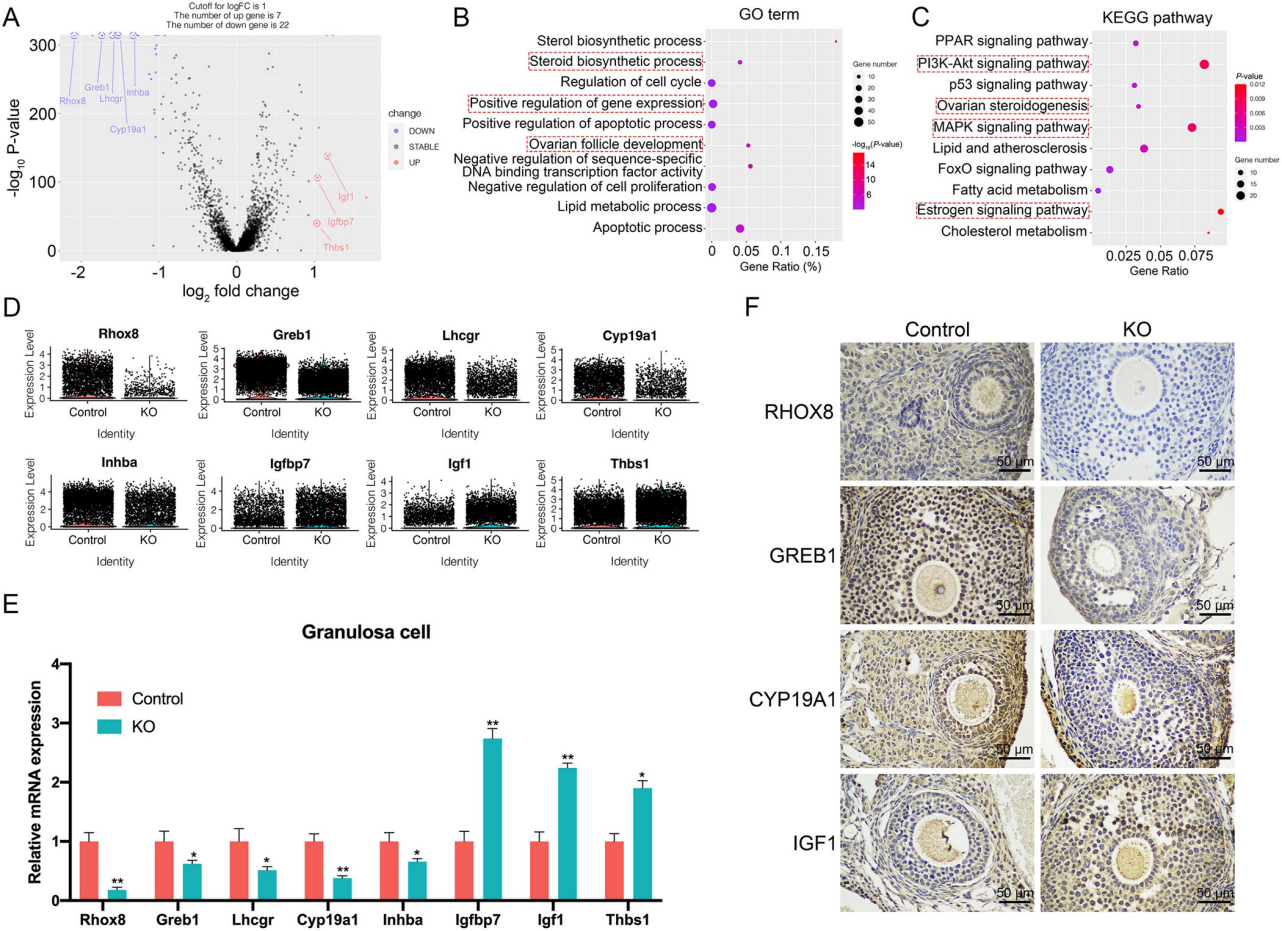
Analysis of DEGs of granulosa cells between control and KO groups. (A) Volcano plot of genes differentially expressed in granulosa cells between control and KO groups. (B) Bubble chart shows the GO enrichment results of differentially expressed genes in granulosa cells between control and KO groups. (C) Bubble chart shows the KEGG enrichment results of differentially expressed genes in granulosa cells between control and KO groups. (D) Vlnplots of the expression level of representative DEGs in granulosa cells between control and KO groups. (E) RT-qPCR validation of representative DEGs in granulosa cells between control and KO groups. (F) IHC validation of representative DEGs in granulosa cells between control and KO groups. *p < 0.05, **p < 0.01.

To investigate the consequences of ERα ablation on granulosa cell function, we assessed the DEGs in granulosa cells derived from control and KO mice. A volcano plot analysis confirmed that the transcriptome difference between these two groups was characterized by 7 significantly upregulated and 22 significantly downregulated genes with a |log2(FC) | > 1 (Fig 5A and S3 Table). Gene ontology analysis revealed that the most significantly differentially regulated pathways between the control and KO groups were related to steroid biosynthetic process, positive regulation of gene expression and ovarian follicle development (Fig 5B). KEGG-enriched analyses were used to identify ongoing bioprocesses mainly in granulosa cells. The results showed that “PI3K-AKT signaling pathway”, “ovarian steroidogenesis” and “estrogen signaling pathway” were mostly represented in granulosa cells (Fig 5C). By using violin plots, we found that the expression of *Rhox8*, *Greb1*, *Lhcgr*, *Cyp19a1* and *Inhba* was significantly downregulated in KO granulosa cells. In contrast, the expression of *Igfbp7*, *Igf1* and *Thbs1* was significantly upregulated in KO granulosa cells (Fig 5D). To validate the genes with significant changes in expression identified within the single-cell sequencing dataset, we performed RT-qPCR on granulosa cells and immunohistochemical staining (IHC) from control and KO mice ovary. Transcriptional gene and protein expression changes were found to be concordant with the scRNA-seq data confirmed by RT-qPCR and IHC results (Fig 5E, 5F and S3F Fig). Finally, to identify distinct granulosa cells engaging in proliferation and apoptosis, we examined the expression pattern of cell cycle specific genes in control and KO granulosa cells (S3D and S3E Fig). The result showed that the genes related with G2M and S stage had a lower expression level in KO granulosa cells than that in control granulosa cells, suggesting a decreased proliferation rate in KO granulosa cells. These results indicate that ERα knock out in mouse ovary can affect the development and function of granulosa cells by modulating the expression of these DEGs.

We also analyzed the expression patterns of the mesenchymal cell subpopulation in response to ERα knockout. The results indicated significant changes in gene expression, with several genes downregulated in the KO group, including C3, Trf, and Igf1, while others, such as Enpep and Gpc3, were significantly upregulated (S4 Fig). This analysis provides a clearer understanding of ERα’s role in ovarian steroidogenesis and highlights the complex regulatory dynamics within mesenchymal cells.

### GREB1 is induced by ERα binding to ERE upstream of the GREB1 promoter

**Fig 6:**
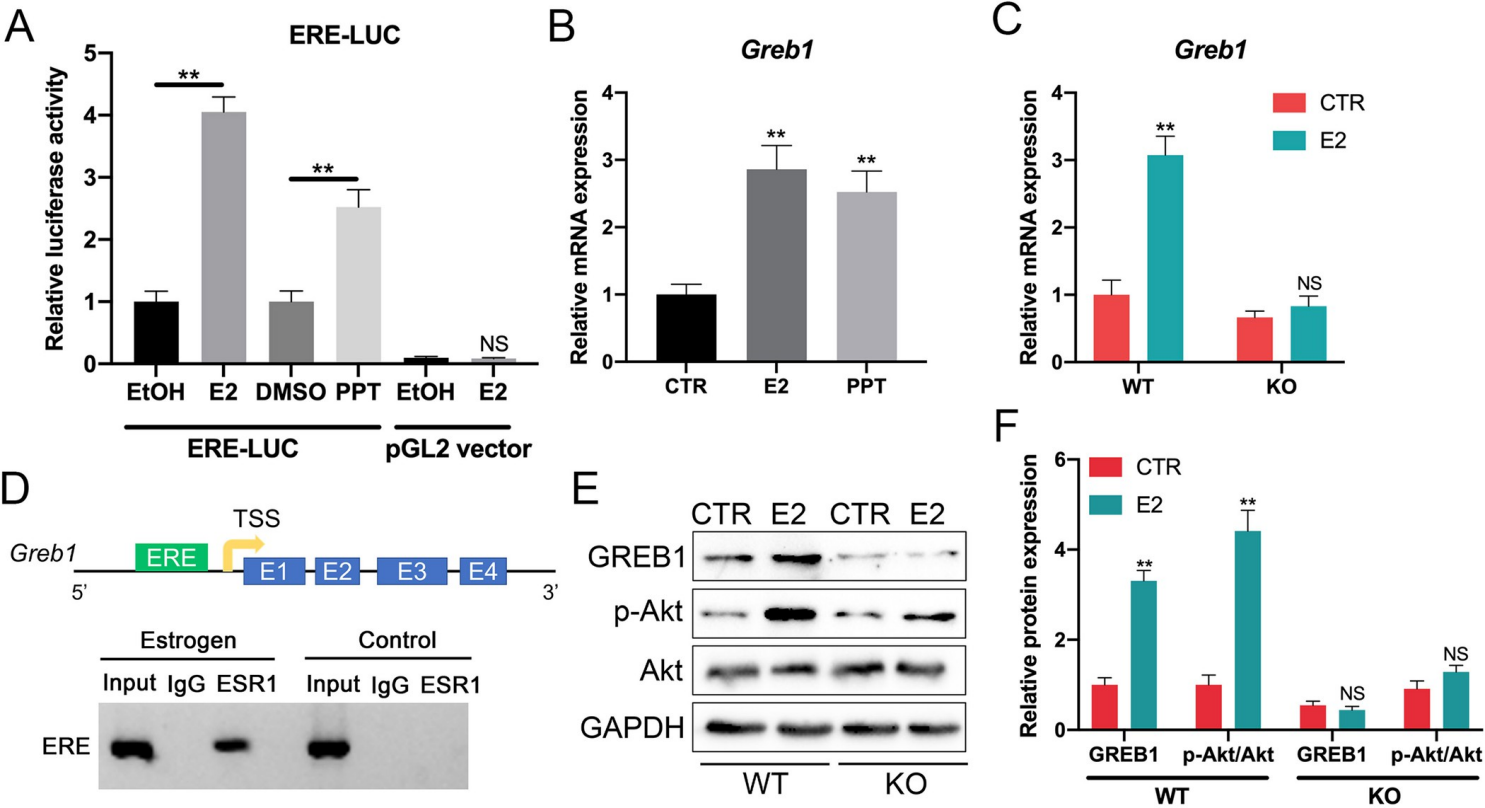
GREB1 is induced by ERα binding to ERE upstream of the GREB1 promoter. (A) Relative luciferase activity of ERE-LUC vector or pGL2 vector (backbone) in granulosa cells treated for 24 h with E2 (10 nM), PPT (10 nM) orrelated vehicles (ethanol and DMSO). (B) Relative expression levels of *Greb1* mRNA in granulosa cells treated with either control vehicle (CTR), E2 (10 nM) or PPT (10 nM). (C) Relative expression levels of *Greb1* mRNA in WT or ERα KO granulosa cells treated for 24 h with either control vehicle (CTR) or E2 (10 nM). (D) Chromatin immunoprecipitation-PCR shows ligand-dependent ESR1 binding to ERE associated with the *Greb1* promoter. (E-F) Western blot shows the protein levels of GREB1 and p-AKT in WT or ERα KO granulosa cells treated for 24 h with either control vehicle (CTR) or E2 (10 nM). *p < 0.05, **p < 0.01.

The functional mechanism of estrogen (17β-estradiol; E2) can be delineated by examining the gene expression changes upon E2 treatment in cells. Microarray analysis revealed *Greb1* as a substantially E2-upregulated gene in tumors derived from an E2-responsive mouse model of ovarian cancer [26]. GREB1 expression is positively correlated with ERα status in breast cancer cell lines and primary breast tumors [27, 28]. In our investigation, we aimed to uncover the potential molecular mechanisms through which ERα may regulate granulosa cells. First, we observed a four-fold increase in luciferase activity following E2 treatment of ERE-LUC-transfected granulosa cells, suggesting that ERα may function via the genomic pathway (Fig 6A). The ERα agonist PPT also elicited a three-fold enhancement in ERE-LUC reporter activity (Fig 6A). Given our findings that E2-bound ERα can transactivate an ERE-luciferase reporter gene, we postulated that *Greb1* expression might be induced by E2 in granulosa cells. Consistent with this hypothesis, E2 treatment of granulosa cells markedly elevated *Greb1* mRNA levels by approximately 3-fold after 6 hours (Fig 6B). The ERα agonist PPT fully recapitulated the E2-induced increase in *Greb1* expression (Fig 6B). Notably, the *Greb1* induction observed after 24 hours of E2 treatment was entirely abolished in ERα KO granulosa cells (Fig 6C). Employing chromatin immunoprecipitation-PCR (ChIP-PCR) to investigate whether *Greb1* transcription is stimulated by ERα binding to the *Greb1* promoter region, we detected ligand-dependent ERα binding to ERE in granulosa cells (Fig 6D). Subsequently, we evaluated GREB1 protein abundance and the activation of the AKT signaling pathway, which was enriched in granulosa cells. Our results demonstrated that E2 treatment substantially increased GREB1 protein levels and AKT phosphorylation in WT granulosa cells. Conversely, ERα ablation led to a marked reduction in GREB1 expression and AKT activation in E2-treated granulosa cells (Fig 6E and 6F). In summary, our findings support the notion that GREB1 abundance can be modulated by ERα in granulosa cells.

## Discussion

Estrogen receptors (ERs) are critical for maintaining normal physiology and reproductive functions. In recent years, significant advances have been made in understanding the molecular mechanisms and biological roles of ERα-mediated responses. Despite this progress, the specific mechanisms by which ERα impacts female reproduction and other physiological processes, leading to therapeutic developments for estrogen receptor-associated diseases, remain to be elucidated. In the present study, we employed single-cell RNA sequencing (scRNA-seq) to comprehensively examine the transcriptome dynamics of wild-type (WT) and ERα knockout (KO) mouse ovaries, as well as the differential alterations in signaling pathways. Through our analyses, we identified seven distinct cell populations, including granulosa cells, mesenchyme cells, macrophages, vascular endothelial cells, oocytes, an unknown cell type, ovarian surface epithelium cells (OSE), and neutrophils, which exhibited dynamic changes during the investigation period. These fluctuations likely represent the diverse cell populations’ proliferation, differentiation, and cell death processes. While some ovarian somatic cells such as mesenchyme, vascular endothelial cells, and immune cells were included in our analysis, due to limited information regarding other ovarian somatic compartments, we primarily focused on oocytes and granulosa cells. Initially, we examined the transcriptional alterations in oocytes resulting from ERα ablation using high-resolution scRNA-seq. Subsequently, we investigated the granulosa cell fate transition following ERα deletion. Moreover, we discovered that Greb1, an ERα-regulated gene potentially involved in estrogen action, is induced by E2 via ERα binding to ERE in mouse granulosa cells, as previously demonstrated in cancer cells [29]. Nevertheless, we observed that ERα ablation impeded GREB1 upregulation and AKT activation by E2 in granulosa cells. It suggested that the ERα-dependent subset of E2 actions may be partially or completely mediated by GREB1, which promotes cell growth and survival in granulosa cell tumors [30]. Therefore, our findings provide a novel perspective on the mechanisms and biological functions of ERα in the ovary, expanding our understanding of its roles in reproductive physiology.

The advancement of efficacious treatments for estrogen-associated disorders hinges on comprehending the physiological roles and mechanistic underpinnings of ERα in both human health and disease states. Previously, investigations into the transcriptomic alterations in ovarian cells were primarily conducted using bulk RNA-seq, which generally yields averaged gene expression values across cell populations [31]. In the present study, we employed scRNA-seq to elucidate the impact of ERα on oocytes and granulosa cells at the transcriptomic level, thereby refining our understanding of the consequences of ERα ablation on specific cell types. Utilizing this advanced technique, we demonstrated that ERα deletion in the ovary substantially impedes oocyte maturation and ovulation progression due to the disruption of steroid metabolic processes. Moreover, we observed marked alterations in the growth status of both oocytes and granulosa cells following ERα disruption. These findings not only underscore the pivotal role of ERα in female reproductive processes but also lay the groundwork for further in-depth investigations, ultimately contributing to the development of targeted therapeutic strategies for estrogen-related diseases.

To thoroughly investigate the transcriptional regulatory mechanisms of oocytes and granulosa cells in the ERα knockout mouse model, we employed various algorithms and bioinformatic analyses. Initially, we utilized existing data to validate the oocyte developmental trajectory following ERα ablation. Intriguingly, we observed that certain ERα-deleted oocytes were redirected to alternative cell fates, displaying a markedly distinct cell state compared to control oocytes. By scrutinizing distinct gene sets along this trajectory, we pinpointed biological processes related to ovarian steroidogenesis and the ovulatory cycle. Furthermore, we identified several DEGs in oocytes that encode essential components for estradiol synthesis and steroidogenesis, both of which are critical for reproductive processes. For instance, *Hsd17b7* predominantly converts estrone to estradiol, particularly during the luteal phase of the rodent ovarian cycle, and is responsible for the final step in estradiol synthesis while being upregulated in ERα KO ovaries [32, 33]. ERα ablation also rapidly elevates the expression of the steroidogenic acute regulatory (*Star*) gene in oocytes, a known luteinization marker gene [34]. *Abcb1b*, which encodes the multidrug-resistant transporter (also referred to as P-glycoprotein), is acutely regulated by CCAAT/Enhancer-Binding Proteins (C/EBP)-α and -β in ovaries and exhibits high expression levels in ERα KO ovaries [35]. Although its specific function in luteal cells remains to be elucidated, it may be involved in cholesterol or progesterone transport. Adult female mice lacking *Ltbp1* exhibit impaired fertility characterized by ovarian cyst formation and reduced estrogen and progesterone levels [23]. *Akr1c18* encodes 20α-hydroxysteroid dehydrogenase (20α-HSD), which converts progesterone into the inactive metabolite 20α-hydroxyprogesterone (20α-OHP) [24]. Piekorz et al. demonstrated that *Akr1c18* deletion in mice results in persistent progesterone production and subsequent parturition failure [36]. Additionally, the absence of a functional SCARB1 protein in female mice leads to morphological abnormalities in ovarian follicular structures, similar to the αERKO phenotype [37]. Another study reported that *Ptgfr* plays a role in maintaining the estrous cycle [34]. Our findings revealed significantly reduced expression of *Ltbp1*, *Akr1c18*, *Scarb1*, and *Ptgfr* in ERα-deficient female mice. Consequently, we deduced that ERα KO female mice exhibit a paucity of preantral and small antral follicles, numerous large and hemorrhagic cystic follicles, and an absence of corpora lutea, which may be attributed to the dysregulation of these genes in the ovary.

The most significant changes in composition and cell states were identified in granulosa cells, particularly within follicles based on their stage of development, reflective of their important role in cyclic follicular maturation and hormone production. Early preantral follicle numbers are considered relatively stable during follicle growth and maturation [38], as they are largely unresponsive to gonadotropins [39]. In contrast, antral follicles exhibit more variability in numbers and size. Our study revealed that the majority of granulosa cells in preantral follicles originated from control mouse ovaries, while those in antral follicles were primarily derived from ERα KO mouse ovaries. Additionally, the corpus luteum cluster was predominantly identified in control granulosa cells. Genes enriched in this cluster have been previously implicated in the ovulatory process and are regulated by the luteinizing hormone (LH) surge, corroborating that ERα disruption results in ovulation and fertility defects in female mice.

Subsequent bioinformatic analyses of the granulosa cell transcriptome identified numerous differentially expressed genes (DEGs) previously reported as essential for steroid biosynthetic processes and ovulation. For instance, *Rhox8* is primarily expressed in mouse ovarian granulosa cells and exhibits peak expression during the periovulatory phase at 8 h post-hCG administration. *Rhox8* is specifically stimulated by the progesterone receptor, suggesting its involvement in LH-dependent follicular rupture by inducing secondary progesterone-regulated genes crucial for ovulation [40]. In ERα KO mouse granulosa cells, *Rhox8* was the most down-regulated gene and may contribute to granulosa cell proliferation, survival, or differentiation. Ovarian follicles lacking FSH or FSH receptors fail to advance to the preovulatory stage, resulting in infertility. A hallmark of preovulatory follicles is the presence of *Lhcgr* on granulosa cells. The PI3K/AKT pathway activation is required for FSH-induced endogenous *Lhcgr* mRNA expression in granulosa cells. However, PI3K/AKT pathway disruption and *Lhcgr* downregulation in ERα-deleted granulosa cells may contribute to ovulation failure and female sterility [41].

Granulosa cells are pivotal in hormone secretion, with *Cyp19a1* playing a significant role in E2 synthesis. *Cyp19a1* expression is regulated by FSH and LH at the mRNA level. CYP19A1 knockdown modulates hormone secretion and cell proliferation in follicular granulosa cells [42]. ERα KO mice exhibit chronically elevated LH, E2, and testosterone due to disrupted negative feedback, with Cyp19a1 significantly decreased in ERα-deleted granulosa cells. Ligand-independent responses are ER-mediated effects observed after activating other pathways, such as insulin-like growth factor 1 (IGF1) receptor-mediated signaling, leading to ER-mediated transcriptional responses independent of estrogenic steroid ligands. Recent studies indicate that IGF1 stimulation can result in ERα recruitment to chromatin, with ChIP-PCR analysis confirming ERα binding to specific ERE sequences of the *Igf1* gene [43, 44]. Our investigation revealed that *Igf1r* was significantly upregulated in ERα-deleted granulosa cells, which may regulate granulosa cell growth and proliferation.

**Fig 7:**
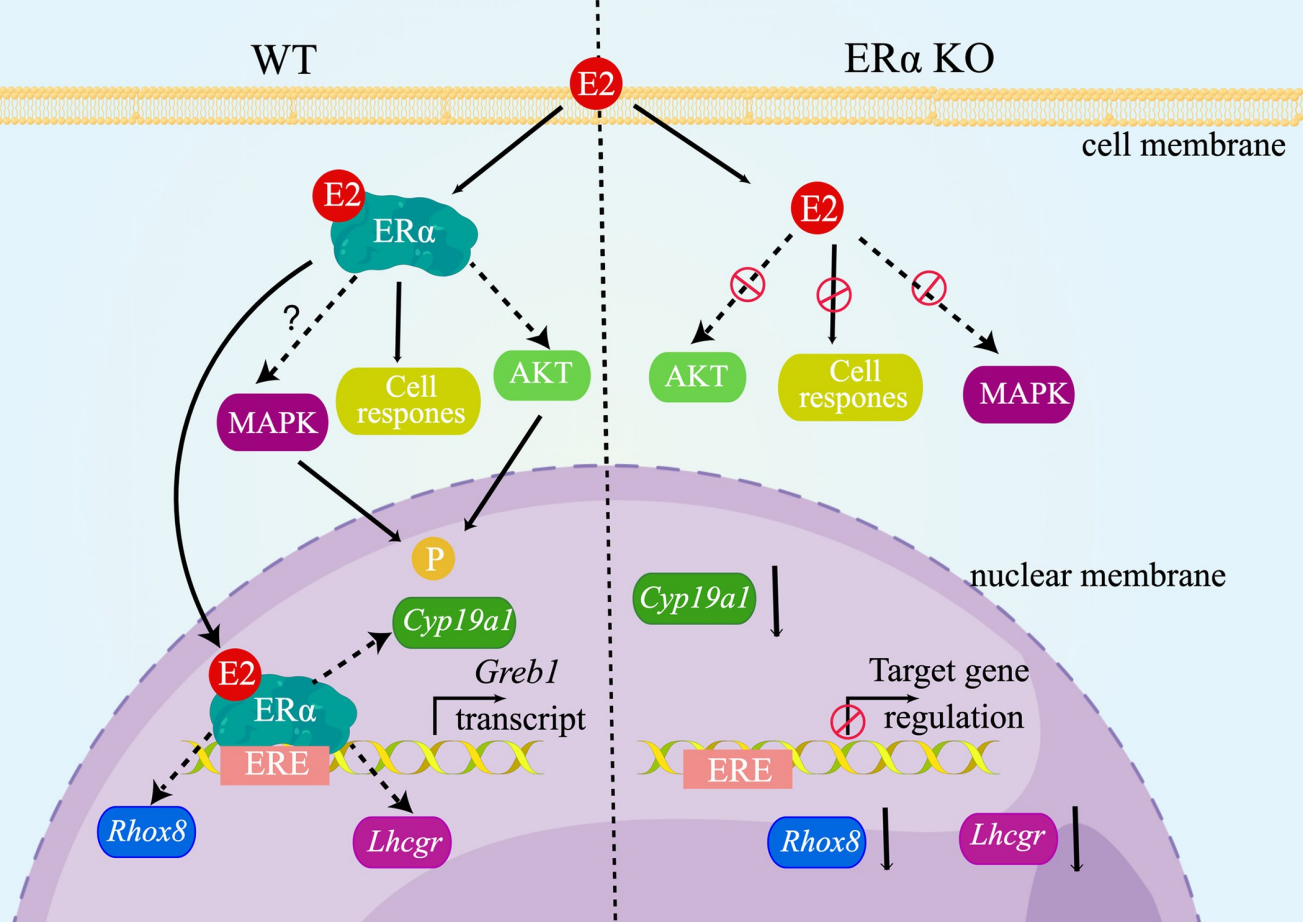
Proposed mechanisms of action of ERα in mouse ovary.

In general, the fundamentals of estrogen response can be deduced from the earlier description of ER domains. Pioneer factors such as FOXA1 facilitate accessibility by binding and partially opening chromatin, enabling ER-ERE interactions at appropriate sites within the cell [45]. The ER DBD associates with ERE motifs in accessible chromatin regions, while the LBD binds E2, initiating conformational changes in the ER protein. This interaction between E2/ER and transcriptional coactivators, including those with chromatin remodeling activities, is subsequently enabled. In the current study, we aimed to unravel the potential roles and mechanisms of ERα action by conducting cell-based and molecular analyses using mouse granulosa cells. Notably, we demonstrated that ERα-mediated activity could occur through a genomic pathway, stimulating the expression of GREB1 (growth regulation by estrogen in breast cancer 1), a crucial regulator of E2-stimulated epithelial ovarian cancer and granulosa cell tumor cell growth (Fig 7). Utilizing reporter assays, we showed that ERα could act through an ERE-dependent mechanism in granulosa cells, as previously reported [46]. This observation was reinforced by the finding that treatment with the ERα agonist PPT upregulated *Greb1* expression, which is necessary for E2-stimulated growth in several hormonally regulated tumors [47]. *Greb1* upregulation was absent in ERα-deficient granulosa cells, confirming the specificity of ERα activity on *Greb1* regulation in these cells. Importantly, our subsequent analyses in granulosa cells revealed that E2-induced *Greb1* expression occurs through ERα binding to EREs, as confirmed by ChIP-PCR. Membrane interactions trigger rapid signaling responses (excluding transcriptional components), including activation of intracellular signaling pathways such as AKT. This mechanism appears to play a significant role in peripheral E2 cellular responses. Consequently, our study demonstrated that signaling pathways like PI3K-AKT and are essential for interactions between oocytes and granulosa cells. ERα knockout markedly reduced GREB1 production and AKT activation in E2-treated granulosa cells. We therefore hypothesize that dysregulated genes following ERα knockout in mouse ovaries could offer therapeutic targets to alleviate infertility caused by ERα inactivation.

## Conclusion

In conclusion, through the application of scRNA-seq, we have uncovered the transcriptional dynamics of various ovarian cell types, particularly oocytes and granulosa cells, following ERα disruption in female mice. Our findings highlight that ERα deletion may result in impaired ovulatory potential due to the dysregulation of multiple genes, which are potentially essential for steroid biosynthesis and ovulation processes. Furthermore, ERα deletion impacts the proliferation and function of granulosa cells, which play a critical role in oocyte development regulation. In future studies, the use of ERα conditional knockout models, such as ERα oocyte-specific or granulosa-specific knockouts, could provide more detailed insights into the cell-type-specific roles of ERα. These models are likely to exhibit distinct phenotypes, such as impaired oocyte maturation and ovulation defects, given the critical role of ERα in oocyte-granulosa cell crosstalk during follicular development. These investigations build upon our earlier morphological characterizations of the αERKO ovary and offer additional evidence that the most pronounced ovarian phenotypes in the αERKO arise from substantial transcriptomic alterations. However, given the physiological differences between mice and humans, murine models may not accurately represent human diseases. As such, a more comprehensive understanding of the role and mechanisms of ERα in female reproduction could be achieved by examining samples from individuals with ERα-related disorders. Moving forward, we plan to utilize ovarian tissue samples from patients with ERα inactivation to further elucidate the reproductive mechanisms of ERα, ultimately providing a more in-depth theoretical foundation for the prevention and treatment of reproductive health issues caused by ERα mutations in humans.

## Supporting information

S1 Fig ScRNA-seq of ovaries from control and αERKO mice. (A) InDROP libraries were sequenced, demultiplexed, normalized, and analyzed using the Seurat package in ‘R’. The processed samples clustered into 14 clusters. (B) Heatmap of top 5 markers per cluster ordered by logFc values. (C) The percentage of cell counts for each cell type in the control and KO samples. (D) Heatmap of top 10 highly expressed genes per cluster ordered by logFc values. (E) ERα depletion was verified in the knockout ovaries by Western blot. (F) The protein levels of Hsd17b7, Star, and Plin4 were detected by Western blot.
(TIF)

S2 Fig Dissecting oocytes subclusters. **(A)** Heatmap of top 5 highly expressed genes per cluster ordered by logFc values in oocytes subclusters. **(B-C)** Single-cell pseudotime developmental trajectory of oocytes, which are colored according to two groups (B) and time (C). **(D)** UMAP plot inferring the cell-cycle phase based on expression of a large set of G2/M- and S-phase genes in oocytes. **(E)** Percentages of oocytes in different cell-cycle phases from control and KO mice.
(TIF)

S3 Fig Dissecting granulosa cells subclusters. **(A-B)** Single-cell pseudotime developmental trajectory of granulosa cells, which are colored according to two groups (A) and time (B). **(C)** Monocle analysis on subclustering of granulosa cells. **(D)** UMAP plot inferring the cell-cycle phase based on expression of a large set of G2/M- and S-phase genes in granulosa cells. **(E)** Percentages of granulosa cells in different cell-cycle phases from control and KO mice. **(F)** Statistical results of IHC expression in Fig 5F.
(TIF)

S4 Fig Dissecting mesenchyme cells subclusters. **(A-B)** UMAP plot featuring the different cell subclusters belonging to the mesenchyme cells cluster (A). UMAP diagram of the mesenchyme cells subpopulations of control and KO groups (B). **(C)** Proportion of the seven cell subclusters of the mesenchyme cells in control and KO groups. **(D-E)** Single-cell pseudotime developmental trajectory of mesenchyme cells, which are colored according to cell development state. **(F)** Volcano plot of genes differentially expressed in mesenchyme cells between control and KO groups. **(G)** Bubble chart shows the GO enrichment and KEGG pathway results of differentially expressed genes in mesenchyme cells between control and KO groups. **(H)** Vlnplots of the expression level of representative DEGs in mesenchyme cells between control and KO groups.
(TIF)

S1 Table Primers used in this study. (XLSX)

S2 Table DEGs in oocytes. (XLSX)

S3 Table DEGs in granulosa cells. (XLSX)
