# Supplementary figures and images for: Single-cell sequencing reveals transcriptional dynamics regulated by ERα in mouse ovaries
Source: PLoS One. 2024 Nov 21;19(11):e0313867. doi: 10.1371/journal.pone.0313867 (PMC11581351; doi:10.1371/journal.pone.0313867)

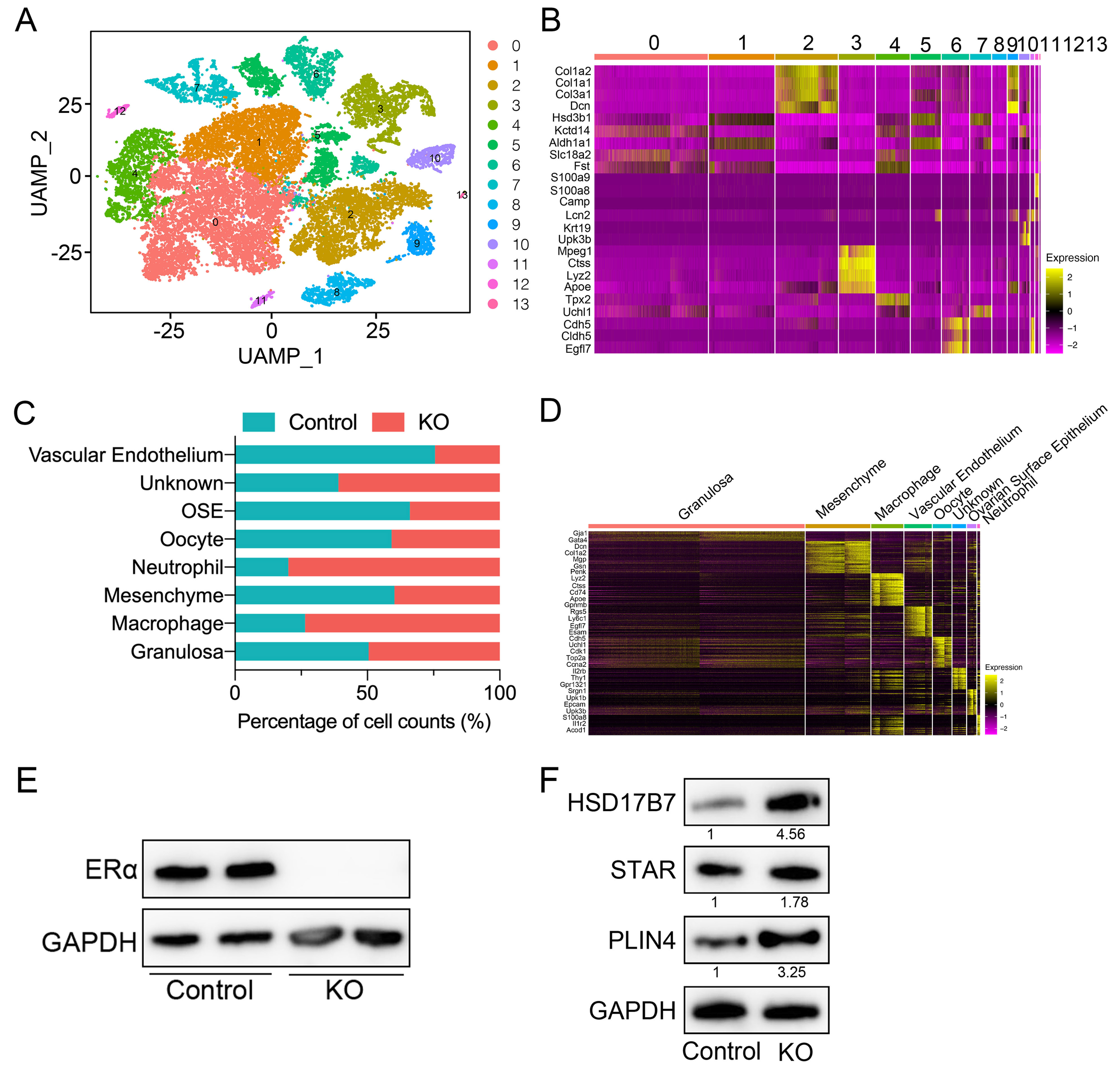

Supplement: S1 Fig — (A) InDROP libraries were sequenced, demultiplexed, normalized, and analyzed using the Seurat package in ‘R’. The processed samples clustered into 14 clusters. (B) Heatmap of top 5 markers per cluster ordered by logFc values. (C) The percentage of cell counts for each cell type in the control and KO samples. (D) Heatmap of top 10 highly expressed genes per cluster ordered by logFc values. (E) ERα depletion was verified in the knockout ovaries by Western blot. (F) The protein levels of Hsd17b7, Star, and Plin4 were detected by Western blot. (TIF) [file pone.0313867.s001.tif]

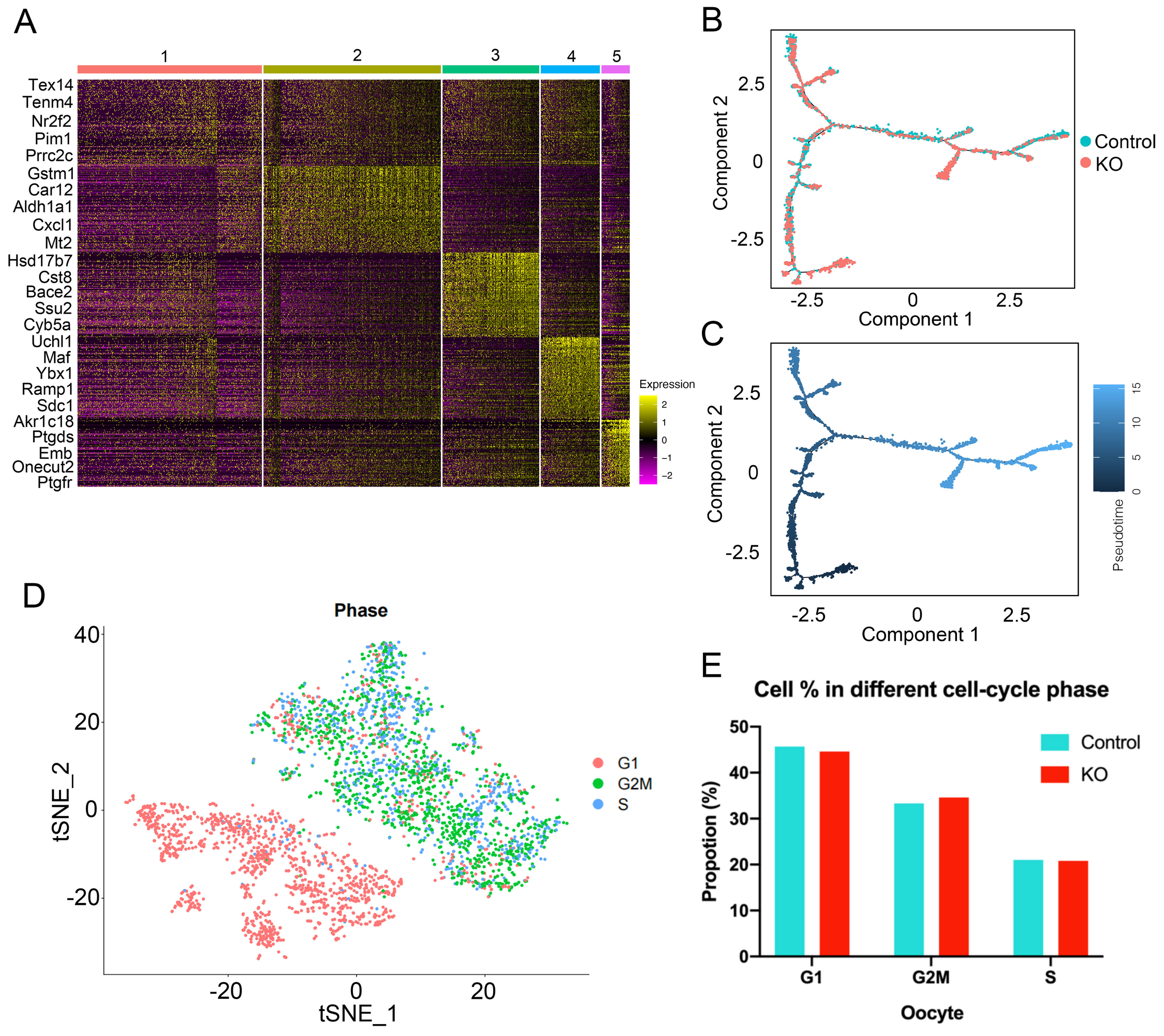

Supplement: S2 Fig — (A) Heatmap of top 5 highly expressed genes per cluster ordered by logFc values in oocytes subclusters. (B-C) Single-cell pseudotime developmental trajectory of oocytes, which are colored according to two groups (B) and time (C). (D) UMAP plot inferring the cell-cycle phase based on expression of a large set of G2/M- and S-phase genes in oocytes. (E) Percentages of oocytes in different cell-cycle phases from control and KO mice. (TIF) [file pone.0313867.s002.tif]

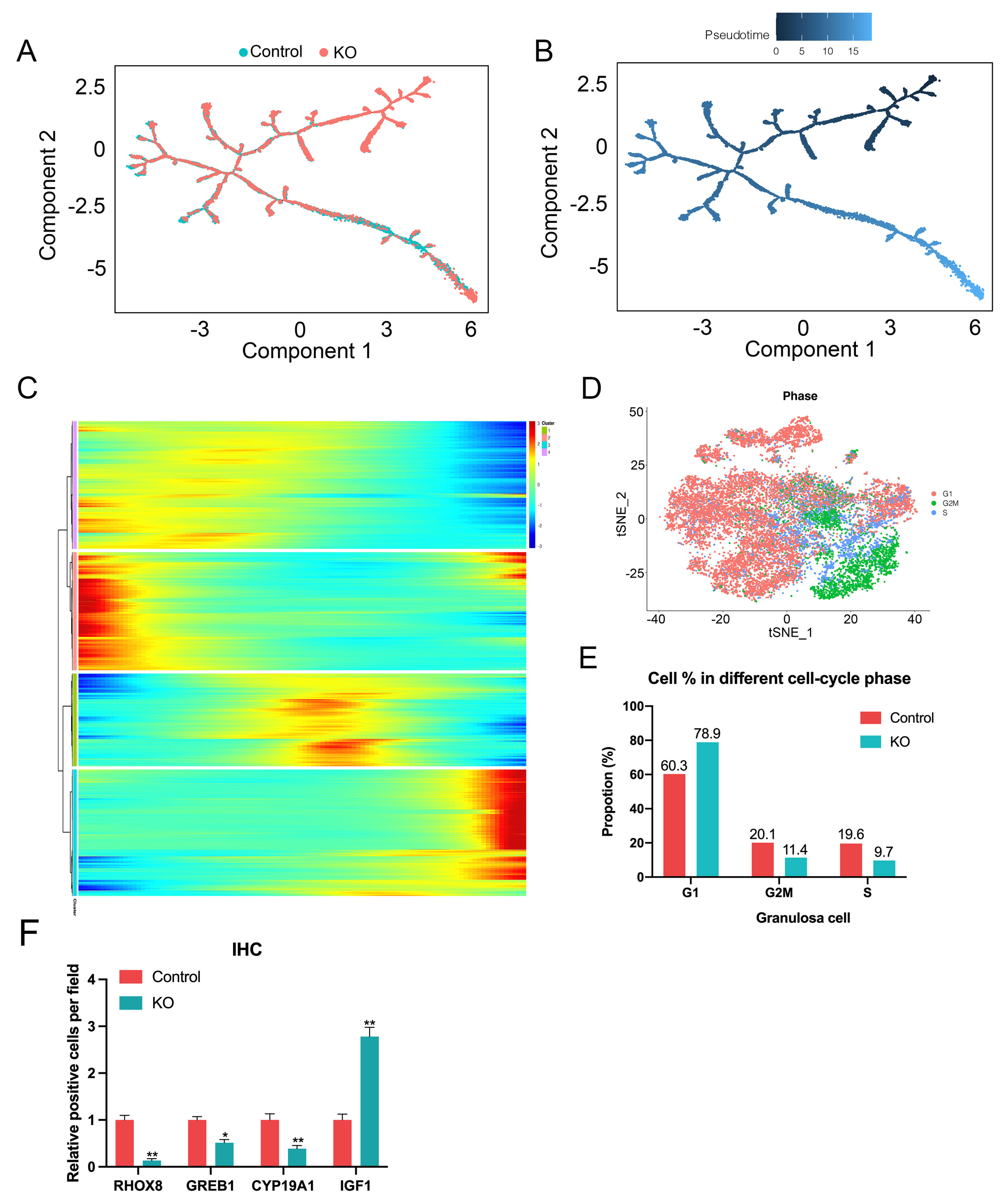

Supplement: S3 Fig — (A-B) Single-cell pseudotime developmental trajectory of granulosa cells, which are colored according to two groups (A) and time (B). (C) Monocle analysis on subclustering of granulosa cells. (D) UMAP plot inferring the cell-cycle phase based on expression of a large set of G2/M- and S-phase genes in granulosa cells. (E) Percentages of granulosa cells in different cell-cycle phases from control and KO mice. (F) Statistical results of IHC expression in Fig 5F. (TIF) [file pone.0313867.s003.tif]

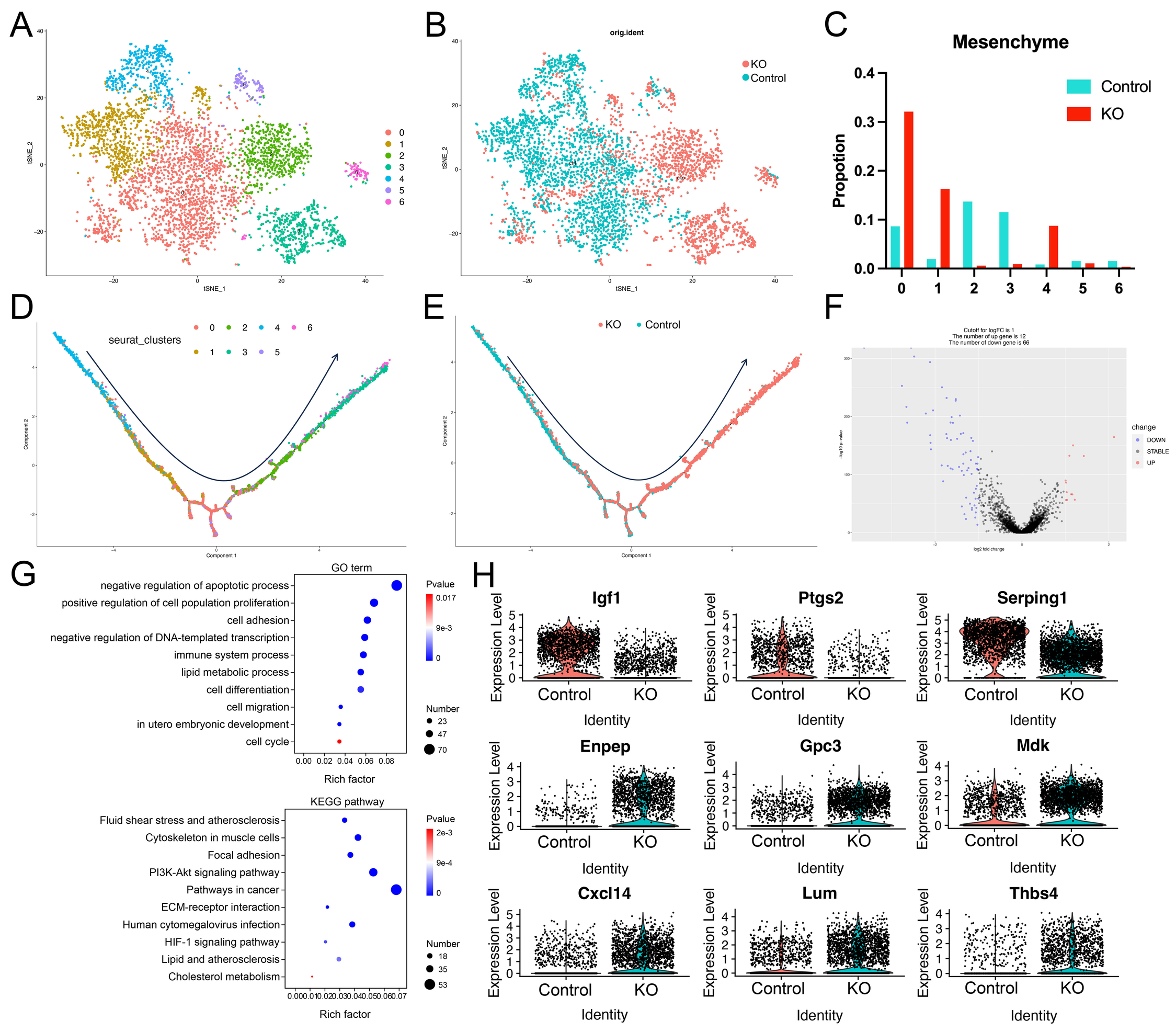

Supplement: S4 Fig — (A-B) UMAP plot featuring the different cell subclusters belonging to the mesenchyme cells cluster (A). UMAP diagram of the mesenchyme cells subpopulations of control and KO groups (B). (C) Proportion of the seven cell subclusters of the mesenchyme cells in control and KO groups. (D-E) Single-cell pseudotime developmental trajectory of mesenchyme cells, which are colored according to cell development state. (F) Volcano plot of genes differentially expressed in mesenchyme cells between control and KO groups. (G) Bubble chart shows the GO enrichment and KEGG pathway results of differentially expressed genes in mesenchyme cells between control and KO groups. (H) Vlnplots of the expression level of representative DEGs in mesenchyme cells between control and KO groups. (TIF) [file pone.0313867.s004.tif]
